# Induction of apoptosis and necrosis in human acute erythroleukemia cells by inhibition of long non-coding RNA PVT1

**DOI:** 10.22099/mbrc.2018.29081.1316

**Published:** 2018-06

**Authors:** Mahsa Salehi, Mohammadreza Sharifi

**Affiliations:** Department of Genetics and Molecular Biology, School of Medicine, Isfahan University of Medical Sciences, Isfahan, Iran

**Keywords:** Long non-coding RNA, LncRNA PVT1, Apoptosis, Acute erythroleukemia

## Abstract

Recent advances in molecular medicine have proposed new therapeutic strategies for cancer. One of the molecular research lines for the diagnosis and treatment of cancer is the use of long non-coding RNAs (LncRNAs) which are a class of non-coding RNA molecules longer than 200 base pairs in length that act as the key regulator of gene expression. Different aspects of cellular activities like cell growth, proliferation, differentiation, apoptosis and migration are regulated by lncRNAs. In various cancers, aberrant expression of lncRNAs has been reported. One of the lncRNAs that showed upregulation in human acute myeloid leukemia (AML) is lncRNA plasmacytoma variant translocation 1 (PVT1). Here, we performed blockage of lncRNA PVT1 in human acute erythroleukemia (AEL) cell line (KG1) using antisense LNA GapmeRs. Then, at different time points (24, 48 and 72 hours) after transfection, qRT‑real‑time PCR and AnnexinV/Propidium Iodide staining assay were performed. The data were processed using the ANOVA test. At all three time points, the ratio of apoptotic cells in the PVT1 antisense LNA GapmeRs treated group was higher than the other groups. The ratio of necrotic cells in the antisense LNA GapmeRs group was also higher than the other groups. These assessments show that inhibition of lncRNA PVT1 could significantly induce apoptosis and necrosis in KG1 cells. Our findings can be used in translational medicine for future investigation in acute erythroleukemia and treatment approach based on antisense therapy.

## INTRODUCTION

Recent advances in sequencing technologies have revealed new secrets of the non-coding RNA world [1]. Long non-coding RNAs (LncRNAs) are defined as endogenous cellular RNA molecules, more than 200 base pairs in length, which have lost their protein-coding capacity and act as a key regulator of gene expression in transcriptional and post-transcriptional levels[1, 2]. Similar to messenger RNAs (mRNAs), lncRNAs are usually transcribed by RNA polymerase II (Pol II), spliced, and mostly polyadenylated [2]. Although the biological function of the majority of lncRNAs has not yet been discovered, emerging evidence indicates that lncRNAs are involved in the various biological process including DNA replication, transcription, alternative splicing, translation, stem cell pluripotency, proliferation, apoptosis [3]. 

Dysregulation of lncRNAs, including both down- and up-regulation of them is strongly associated with the pathogenesis of diverse human diseases including heart and cardiovascular diseases, metabolic, inflammatory, infectious, auto-immunity, neurodegenerative disorders and cancers [4]. Acute erythroleukemia (acute myeloid leukemia M6, or M6 AML, according to the classification of French, American, British: FAB) is an uncommon type of acute myeloid leukemia with poor prognosis characterized by a predominant erythroid proliferation [5]. At the moment, due to the lack of understanding of AEL pathogenesis mechanisms, there are no therapeutic agents that target specific pathways or molecules in this disease and similar to other AML cases, patients with AEL are also treated by chemotherapy and bone marrow transplantation [6, 7]. However, induction failure, relapse, and toxicity of chemotherapeutic agents are the most important issues during treatment of patients with AEL, reflecting a need for the development of novel and specified therapeutic options [6]. LncRNAs, as influential players in cell function, can potentially shed light on the pathogenesis of AEL and lead to the development of novel therapeutic regiments [8]. 

Recently, overexpression of lncRNA plasmacytoma variant translocation 1 (PVT1) in AML is reported [9]. PVT1, one such oncogenic lncRNA, is a large locus located 57 kb downstream of MYC *in the* well-known cancer-related region 8q24 [10]. Increased copy number and overexpression of PVT1 have been revealed in many types of human malignancies including bladder cancer, gastric cancer, colorectal cancer, ovarian cancer and breast cancer [11-14]. PVT1 mediates increased cell proliferation, decreased apoptosis and chemotherapy resistance in these cancers [15]. Based on these findings, we proposed that degradation of lncRNA PVT1 is of therapeutic effect in AML. Several methods have been examined for inhibition of lncRNAs [16]. Antisense LNA GapmeRs is a new generation of antisense oligonucleotides consist of a DNA stretch flanked by locked nucleic acid (LNA) nucleotides. These single strand antisense oligonucleotides induce degradation of target sequence via RNase H-dependent mechanism [16, 17]. In the present study, we have used antisense LNA GapmeRs to inhibit lncRNA PVT1 in acute erythroleukemia (AEL, also known as AML M6) cell line (KG-1) and its effects on the apoptosis and necrosis of KG-1 cells were assessed.

## MATERIALS AND METHODS

**Cell culture: **KG1 cell line (human acute erythroleukemia, AML M6) was purchased from National Cell Bank of Iran (Pasteur Institute, Tehran, Iran). Cells were cultured in RPMI 1640 (Gibco, Paisley, UK) medium supplemented with 10% of fetal bovine serum (FBS; Gibco, Paisley, UK), 100 U/ml of penicillin and 100 mg/ml of streptomycin (Sigma-Aldrich, Saint Louis, MO, USA). The cells were grown at 37^o^C in a humidified incubator with 5% CO_2_. To maintain the exponential phase, cells were passaged two times per week.

**Cell transfection** **:** The lncRNA PVT1 sequence was obtained from a reputable site: http://www.Incrnadb.org (accession id:NR_003367.2). Antisense LNA GapmeRs and antisense LNA GapmeR Negative controls (ALGNC) oligonucleotides for lncRNA PVT1 were purchased from the Exiqon (Copenhagen, Denmark). Antisense LNA GapmeRs and ALGNC were labeled at their 5’ ends with a fluorescent dye, 6-FAM (6-carboxyfluorescein). KG1 cell transfection was performed using the PolyFect™ transfection reagent kit (Qiagen, Hilden, Germany) according to the manufacturer's instructions. Briefly, 5×10^5^ cells, in the exponential growth phase, were seed into six-well culture plates (Nunc, Roskilde, Denmark) containing 1.8 ml RPMI 1640 per well without antibiotic and FBS. Six picomoles of antisense LNA GapmeRs was mixed with 12 μl of Polyfect™ in 300 μl of Opti-MEM I Medium (Gibco, Paisley, UK) and subsequently incubated at room temperature for 10 min. Then, the complex was added to the cells and rotated cautiously to ensure even distribution over the entire plate surface. After 6 h of incubation, FBS and antibiotics were added to the cells and then the cells were incubated for 24, 48 and 72 h. Untreated cells and cells transfected with ALGNC were cultured in parallel to antisense LNA GapmeRs transfected cells. Antisense LNA GapmeRs was conjugated with 6-FAM fluorescein and KG1 Cells transfected with antisense LNA GapmeRs were seen by a fluorescence microscope.

**Reverse Transcriptase lncRNA PVT1 Real-Time PCR:** Reverse transcriptase (RT) lncRNA real-time PCR was performed to determine the efficiency of lncRNA PVT1 inhibition by antisense LNA GapmeRs. Briefly, total RNA was extracted with miRCURY RNA Isolation Kit (Exiqon, Copenhagen, Denmark) at 24, 48 and 72 h after transfection. RNA concentration and purity were measured at an OD of 260 to 280 nm with Spectrophotometer (Bio-Tek Instruments, Winooski, VT, USA). Then, the isolated total RNA was reverse transcribed into complementary DNA (cDNA) using the Universal cDNA Synthesis Kit (Exiqon, Copenhagen, Denmark). Real-time PCR was performed using the SYBR Green master mix Kit (Exiqon, Copenhagen, Denmark) and specific lncRNA PVT1 primers (all consumables in this section were from Exiqon, Copenhagen, Denmark). The ABI Step One Plus (ABI, USA) instrument was used for real-time PCR experiments and the ΔΔCt method was used for data calculation.

**Apoptosis and necrosis assay:** The Annexin-V-FLUOS Staining Kit (Roche, Mannheim, Germany) was used for detection of apoptosis and necrosis in KG1 Cells. For the detection of phosphatidylserine, an apoptosis marker, on the outer leaflet of the apoptotic cell membrane, Annexin-V was used. To differentiate necrotic cells propidium iodide (PI) staining was performed. The procedure was performed at 24, 48 and 72 h after transfection according to the manufacturer’s instruction (untreated cells were used for controls). Concisely, 24, 48 and 72 h after transfection, the KG1 cells which were seeded into six-well plates at a density of 5×10^5^ cells per well were transferred to flow cytometry tubes and centrifuged for 5 min at 1500 rpm. The supernatant was removed away, and cells were washed with 1 ml of cold phosphate-buffered saline (Gibco, Paisley, UK). 100 ml of the prepared solution (based on kit instruction) was added to each tube and incubated at room temperature in the dark for 15 min. After incubation, 300 μl of AVBB solution was added to each tube and analyzed by FACSCalibur flow cytometer (BD, California, USA) with 488 nm excitation, 518 nm band-pass filter for fluorescein-conjugated annexin V detection and a filter>600 nm for PI detection.

**Statistical analysis:** All tests were performed in triplicate and the results were analyzed using SPSS version 22 (IBM, New York, NY, USA) software. Two-way ANOVA (two-way analysis of variance) and also post hoc test to examine were considered. Data presented as mean ± SD. Statistical significance was defined as P<0.05.

## RESULTS

**Figure 1:**
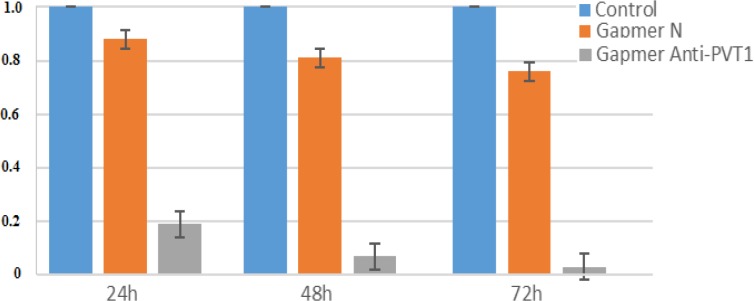
Assessment of the lncRNA PVT1 level by real time PCR, 24, 48, and 72 hours after transfection. The ΔΔCt method was used for data analysis, and the control group was considered as a reference for each time point.

For inhibition of lncRNA PVT1, the antisense LNA GapmeRs was transfected to KG-1 cells with PolyFect™ transfection reagent kit. As the transfected oligonucleotides were fluorochromeconjugated, transfection efficiencies were assessed by fluorescence microscopy. Expression of lncRNA PVT1 was evaluated by reverse transcriptase lncRNA real-time PCR in KG-1 cells transfected with the antisense LNA GapmeRs (LNA GapmeRs group), transfected with antisense LNA GapmeR Negative controls(ALGNC group) and untreated KG-1 cells (untreated groups) at 24, 48 and 72 h after transfection**.** Although lncRNA PVT1expression was a little lower in the ALGNC group compared to the untreated groups, the differences were not statistically significant. However, in all three time points, the expression of lncRNA PVT1was considerably lower in the LNA GapmeRs group compared to the control groups(P<0.001). The greatest level of lncRNA PVT1 expression was at 24 h after transfection in KG-1 cells and then steadily decreased at 48 and 72 h after transfection (Fig. 1).

**Figure 2:**
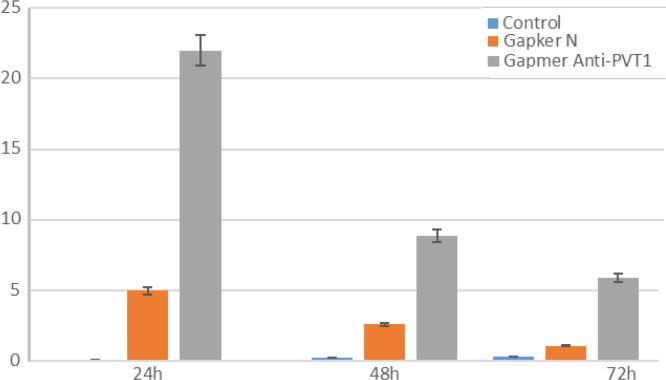
Assessment of apoptosis by annexin V-PI staining performed 24, 48, and 72h after transfection

**Figure 3:**
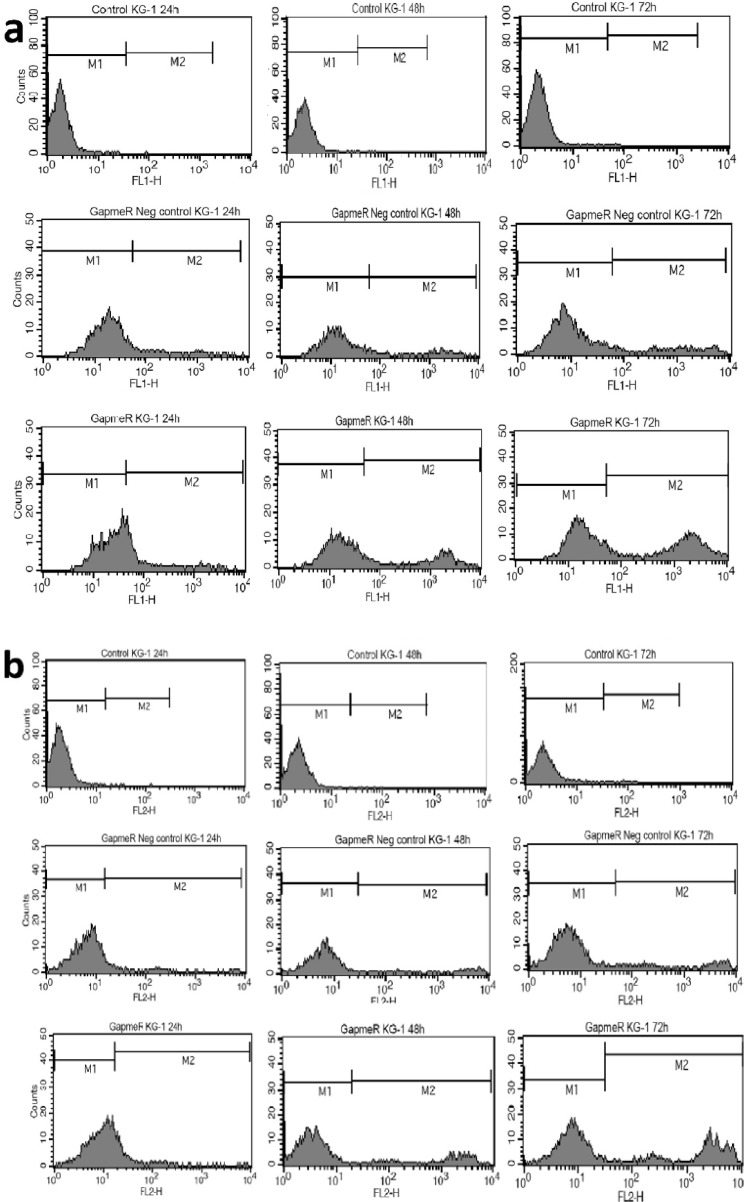
Assessment of apoptosis by annexin-V− propidium iodide staining performed at 24, 48 and 72 h after transfection. Representative cytofluorometric graphs are shown (**a**). Evaluation of necrosis by annexin V–propidium iodide staining performed 24, 48 and 72 h after transfection. Representative cytofluorometric graphs are shown (**b**).

**Figure 4:**
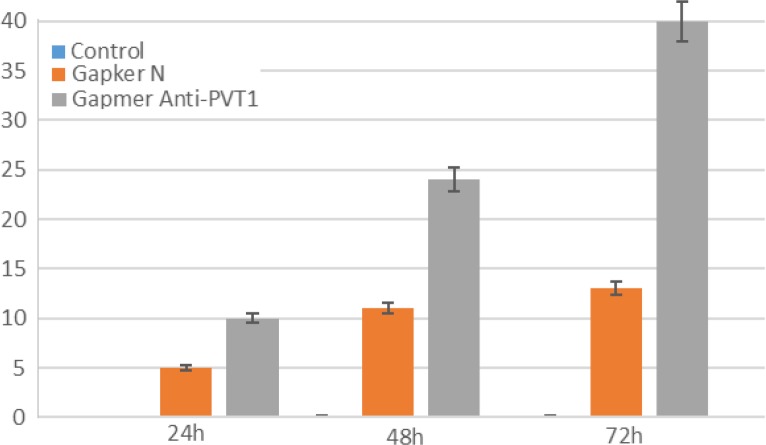
Assessment of necrosis by annexin V- PI staining performed 24, 48, and 72h after transfection

To evaluate the effect of lncRNA PVT1 inhibition on apoptosis and necrosis, cells were stained with annexin-V and PI at 24, 48, and 72 h after transfection. Apoptotic cells were almost undetectable in untreated cells. However, the ratio of these cells minimally increased at 48 and 72 h after ALGNC transfection compared with untreated cells. At all three-time points, the survey of apoptotic assay exhibited a remarkable increase in the apoptotic cells after transfection with antisense LNA GapmeRs compared with ALGNC transfected cells and untreated cells (P<0.001). The highest amount of apoptosis was observed at 24 h post transfection whereas, this ratio was minimally decreased at 48 and 72 h posttransfection. Generally, the results revealed that blockage of PVT1by transfection of antisense LNA GapmeRs markedly promoted the apoptosis of KG-1 cells (Fig. 2, 3).In line with the data of the apoptosis assay, necrosis in the untreated cells was undetectable but the ratio of the necrotic cells minimally increased 48 and 72 h after ALGNC transfection compared to the untreated cells. There was an obvious increase in the necrosis ratio in the antisense LNA GapmeRs transfected cells compared with other groups in all three-time points. The lowest amount of necrosis was seen at 24 h posttransfection whereas, this ratio was minimally increased at 48 and 72 h posttransfection. Altogether, this result indicated that inhibition of PVT1 using antisense LNA GapmeRs could promote the necrosis of KG-1 cells (P<0.001; Fig. 3, 4).

## DISCUSSION

In this study, we used antisense LNA GapmeRs to degrade lncRNA PVT1 in an AEL (AML M6) cell line. Real-time PCR data confirmed that this lncRNA was almost entirely downregulated after antisense LNA GapmeRs transfection. The apoptosis/necrosis assay indicated that PVT1 antisense LNA GapmeRs transfection dramatically increased apoptosis and necrosis. Furthermore, we appreciated that our annexin-V/PI staining method cannot discriminate between late apoptosis and necrosis. However, our results show that degradation of lncRNA PVT1 can increase the apoptosis and necrosis of AEL cells and this is at least partially due to the induction of apoptosis. PVT1 is a candidate oncogenic lncRNA that play important roles in initiation and progression of various malignancies by affecting apoptosis, proliferation and cell cycle [15, 18]. In AML, the lncRNA PVT1 increases. In addition, it is reported that this lncRNA positively regulates C-MYC and leads to increased proliferation of myeloid cells [9].

AML is a clonal heterogeneous disorder with high morbidity and mortality [19]. Despite recent progress, the current therapeutic options for this disease are less than optimal and therefore, the development of new therapeutic options is a necessity [20]. There are increasing studies on oncogenic lncRNAs as an approach to cancer treatment and much effort has been made for treatment of cancer using lncRNA inhibitors [16, 21, 22]. Antisense LNA GapmeRs is one of the methods for efficient inhibiting of oncolncRNAs [17]. In this survey, specified blockage of lncRNA PVT1 using antisense LNA GapmeRs led to induction of apoptosis in KG1 cells and it seems that it is an oncolncRNA in AEL. Given that the increase in apoptosis and necrosis in uncontrolled proliferating cells is an important therapeutic goal in cancer, perhaps lncRNA PVT1 inhibition using antisense LNA GapmeRs technology, could have a potential to treat AEL [23]. Furthermore, in many patients with AML, chemotherapy drugs have no effects and cannot decrease proliferative cells [24]. Many endeavors have been initiated for treatment of cancer using oncolncRNA-inhibitors accompanied by chemotherapy drugs [25]. This study offers that PVT1 antisense LNA GapmeRs can be used alone or in combination with chemotherapy drugs for treating AEL that is resistant to treatment. Furthermore, we realize that further in vivo studies are definitely required to assess the feasibility of this strategy. However, efficient in vivo delivery of anti-lncRNA oligonucleotides remains an obstacle to be tackled before we can move to clinical trials.
